# Effects of Gabapentin Suspension and Rectal Acetaminophen on Postoperative Pain of Adenotonsillectomy in Children 

**DOI:** 10.22038/ijorl.2020.38811.2283

**Published:** 2020-07

**Authors:** Soudabeh Haddadi, Shideh Marzban, Arman Parvizi, Shadman Nemati, Amer Chohdari, Zahra Atrkar Roshan, Hedieh Ramezani

**Affiliations:** 1 *Anesthesia Research Center, Department of Anesthesiology, Alzahra Hospital,Guilan University of Medical Sciences, Rasht, Iran.*; 2 *Rhino-Sinus, Ear, and Skull Base Diseases Research Center, Department of Otolaryngology and Head and Neck Surgery, Amiralmomenin Hospital, School of Medicine, Guilan University of Medical Sciences, Rasht, Iran. *; 3 *Anesthesiologist, Guilan University of Medical Sciences, Rasht, Iran.*

**Keywords:** Acetaminophen, Adenoidectomy, Gabapentin, Pain, Tonsillectomy

## Abstract

**Introduction:**

Adenotonsillectomy is the main treatment for symptomatic tonsillar hypertrophy with postoperative pain as the most common associated complication. The present study aimed to compare the efficacy of gabapentin with that reported for acetaminophen on the postoperative pain of adenotonsillectomy in children.

**Materials and Methods:**

In this double-blind clinical trial, a total of 60 children within the age range of 7-15 years undergoing adenotonsillectomy were randomly allocated to two groups of gabapentin (n=30) and acetaminophen (n=30). The first group received a 10 mg/kg dose of gabapentin suspension preoperatively and placebo (suppository) after the intubation. The second group received a 40 mg/kg dose of acetaminophen suppository and placebo suspension simultaneous with that in the case group. Using the pain scale (based on the score of visual analog scale, prescribed drugs, dosage as well as incidence of postoperative nausea and vomiting were recorded 0, 2, 4, 6, 12, and 24 h after the surgery. The data were analyzed using SPSS software (version 22.0). A p-value less than 0.05 was considered statistically significant.

**Results:**

There was no significant difference between the two groups considering the pain scores at 0, 2, 4, 6, 12, and 24 h after the surgery. In addition, the trend of pain intensity statistically decreased within 0 to 24 h in both acetaminophen (P<0.001) and gabapentin (P<0.001) groups. No statistically significant difference was observed between the two groups regarding the postoperative incidence of nausea and vomiting.

**Conclusion:**

The obtained results of the present study showed that the administration of gabapentin and acetaminophen significantly reduced the postoperative pain of tonsillectomy and need for narcotics in these patients after the surgery. However, in this study, there was no particular reported superiority for any of the two groups in terms of the effects of gabapentin suspension and rectal acetaminophen.

## Introduction

Adenotonsillectomy is one of the most common surgeries performed among children. Tonsillectomy is reported with several complications, such as nausea, vomiting, bleeding, and postoperative pain, with the latter as the most common complication (1).

Generally, the potential complications of tonsillectomy cause difficulty in postoperative management (2). Pain is mainly known as the primary source of morbidity following adenotonsillectomy (3). 

In addition, it is even reported that dehydration or uncontrolled pain is the most common cause of the referral of children to emergency departments after adenotonsillectomy, rather than postoperative bleeding (4). Accordingly, the most important complication of adenotonsillectomy is postoperative pain. 

Adenotonsillectomy-related pain has been reported in 20-50% of children who underwent surgery (5). If the postoperative pain is not well-controlled, it can result in a longer recovery, delayed discharge, and nutritional deficiency leading to dehydration, especially in children. These factors will increase the duration of hospital stay and need for intravenous (IV) fluids (1). Postoperative pain causes more adverse effects in children than those reported for adults. In developing countries, the incidence of pain is higher, and despite the access to the cost-effective methods of pain control, children face the risk of acute and chronic pain (6). Numerous studies have been designed to assess the analgesic effects of several drugs before and after surgery. Nonsteroidal anti-inflammatory drugs (NSAIDs), acetaminophen, opioids, ketamine, dextromethorphan, and topical analgesics are considered for the prevention of pain following adenotonsillectomy (7,8). Sufficient pain control is essential to ensure the quick return to oral intake and hospital discharge; nevertheless, analgesics used in the postoperative period can increase the risk of complications (2). The administration of an effective analgesic that does not cause to increase the risk of bleeding, respiratory depression, nausea, and vomiting can be useful in the management of tonsillectomy patients. Gabapentin might also have the aforementioned properties (9). Gabapentin is a gabapentinoid with the structural analog of gamma-aminobutyric acid functioning as an anticonvulsant. The results of several recent studies associated with the use of gabapentin as an analgesic have been encouraging for postoperative pain of tonsillectomy. In addition, gabapentin has also been proposed as a drug with no increase in the adverse effects after surgery. However, there has been no sufficient evidence to fully support the use of gabapentin in terms of managing the postoperative pain of tonsillectomy (10). With this background in mind, this study aimed to evaluate the effects of gabapentin and acetaminophen suppository on the postoperative pain of adenotonsillectomy.

## Materials and Methods

**Participants**

This double-blinded randomized controlled clinical trial was carried out on a total of 60 children within the age range of 7-15 years with the American Society of Anesthesiologists Physical Status I or II. The subjects were all candidates for elective adenotonsillectomy or adenoidectomy (as the inclusion criterion) admitted to Amir-Al-Momenin Hospital of Guilan University of Medical Sciences, Guilan, Iran, within February 2016 to September 2017. Furthermore, this study was approved by the Ethical Committee of Guilan University of Medical Sciences (code: IR.GUMS. REC. 1395.157). The clinical trial registration code was obtained from the center of registration of clinical trials (2016102115963N7IRCT). After explaining the study objectives and obtaining the informed consent, the patients were entered into the study. The subjects were operated under general anesthesia by a surgeon using the same procedure. The exclusion criteria were a body weight of less than 15 kg at the baseline, allergy to acetaminophen, sensitivity to gabapentin, history of seizure, previous administration of gabapentin, inability to understand the pain score of visual analog scale (VAS) (in the opinion of the study staff), renal or hepatic disease, immunosuppression, history of gastric ulceration, indigestion, dehydration through diarrhea and/or vomiting, history of severe asthma (defined as previous steroid treatment or hospital admission), consumption of analgesics during the last 24 h, alcohol or drug abuse, known glucose-6-phosphate dehydrogenase deficiency, diarrhea, bleeding disorders, and surgical duration of above 90 min. 

***Interventions***

The children were divided into two groups of A (gabapentin suspension) and B (acetaminophen suppository) based on quadruple random blocks. At the initiation of the study, the sequences of the blocks were selected by simple random sampling, sealed in the envelopes, and stored at Anesthesiology Research Center. The subjects were assigned into two groups based on the gradual referral of the patients who met the inclusion criteria.

The children, anesthesiologist, and ear, nose, throat surgeon were blinded to the study groups. The anesthesiologist performing the study was unaware of the constituents of the drugs and allocation of the groups. An anesthetist who was responsible for the questionnaires with different parameters was also unaware of group assignment; accordingly, blinding was satisfactorily maintained throughout the study course. In the gabapentin group, the children received a 10 mg/kg dose of gabapentin suspension 2 h before anesthesia, and the placebo (suppository) was used exactly similar in shape, size, and appearance to acetaminophen suppository after the intubation and maintenance of anesthesia. Each suppository was individually wrapped with identical foil. The standard anesthesia method was performed in the control group, similar to the gabapentin group. The children received the placebo gabapentin suspension simultaneous with that in the case group. The amount, appearance, smell, and taste of gabapentin and placebo suspension were identical. Following the intubation and maintenance of anesthesia, a 40 mg/kg dose of acetaminophen suppository was administered to the patients. Perioperative fluid therapy in both groups consisted of dextrose saline (3-5 ml/kg body weight), the anesthesia method was similarly conducted in both groups. All the patients underwent the anesthetic induction after monitoring and preoxygenation with a 2 mic/kg dose of fentanyl, 2-2.5 mg/kg dose of propofol, 0.5 mg/kg dose of atracurium, and 0.02 mg/kg dose of atropine. The tracheal intubation at an appropriate size was carried out using a dose (50-150 µg/kg) of total IV anesthesia propofol and 0.1-0.3 µg/kg dose of remifentanil based on the weight and height of the subject and maintenance of anesthesia. In the postoperative period, the patients’ vital signs and respiratory symptoms were monitored, and hypotension was treated with ephedrine. Furthermore, in case of respiratory depression, the subjects were treated with masks, oxygen therapy, and positive pressure ventilation. 

***Assessments***

Demographic characteristics, weight, duration of surgery, and amount of bleeding in children were recorded and evaluated immediately after the surgery (while being sufficiently aware to respond) in the postanesthesia care unit. Moreover, the data were assessed after 2, 4, 6, 12, and 24 h stay in the ward for nausea, vomiting, prescribed drugs, dose of drugs, and pain severity. Pain severity was recorded based on the VAS (with Wong-Baker Faces Pain Rating Scale) (11) at 0, 2, 4, 6, 12, and 24 h after the surgery. All the data were recorded by an anesthetist.

***Statistical Methods***

The demographic data were presented as the median and range. In addition, the data were presented as means and standard deviations. For the statistical analysis of the quantitative data, the independent t-test and Mann-Whitney U test were used in terms of the normal and nonnormal distribution of data, respectively. Furthermore, for the evaluation of the qualitative data (e.g., gender, nausea, and vomiting status) in the two groups, the Chi-square and Fisher’s exact tests were utilized in case of the normal and nonnormal data, respectively. A p-value less than 0.05 was considered statistically significant. All the statistical procedures were performed in SPSS software (version 22.0).

## Results

The present study was performed on a total of 60 children undergoing adenotonsillectomy or adenoidectomy to compare the effectiveness of gabapentin suspension and acetaminophen suppository in postoperative pain. The mean values of participants’ age were reported as 8.37±2.01 and 10.40±2.84 years in the acetaminophen and gabapentin groups, respectively, with a significant difference (P=0.002). There was a significant difference in the weight of children between the two groups (P=0.006). However, no significant difference was observed in the amount of bleeding and duration of surgery between the two groups (P=0.796 and P=0.653, respectively) (Table.1). 

**Table 1:** Demographic and intra-operative characteristics of two studied groups

	**Group**	**n**	**Mean±standard deviation**	P-value*
Body weight (kg)	Acetaminophen group	30	30.37±4.16	0.006
	Gabapentin group	30	33.60±4.64	
Amount of bleeding (ml)	Acetaminophen group	10	90.00±13.33	0.796
	Gabapentin group	7	88.57±9.00	
Duration of surgery (min)	Acetaminophen group	30	74.83±7.37	0.653
	Gabapentin group	30	75.67±6.91	
Age (year)	Acetaminophen group	30	8.37±2.01	0.002
	Gabapentin group	30	10.40±2.84	

* Mann-Whitney U test

The trend of pain intensity statistically decreased within 0 to 24 h in both acetaminophen (P<0.001) and gabapentin (P<0.001) groups. There was no significant difference between the two groups in terms of the pain scores at the measurement times (P=0.283). 

In addition, no significant difference was observed in the pain scores between the two groups according to the measurement times. Moreover, the trend of pain variations was not significant during the measurement times (P=0.453; Table.2). 

**Table 2:** Pain scores of two studied groups

**Time interval following surgery**	**Gabapentin group**									
	**Mean±standard deviation**	**Median**	**P 25**	**P 75**	**Mean±standard deviation**	**Median**		**P 25**	**P 75**	**P-value**
0 hour	3.20±1.94	3.00	2.00	5.00	2.73 ±1.57	3.00	2.00		4.00	0.356
2^nd ^hour	1.67±0.92	2.00	1.00	2.00	1.23±0.86	1.00	1.00		2.00	0.095
4^th ^hour	1.83±1.15	2.00	1.00	2.00	1.53±1.07	1.50	1.00		2.00	0.309
6^th ^hour	1.33±0.84	1.00	1.00	2.00	1.53±1.07	1.50	1.00		2.00	0.450
12^th ^hour	1.20±1.21	1.00	0.00	2.00	0.93±0.69	1.00	0.00		1.00	0.468
24^th ^hour	0.93±1.26	1.00	0.00	2.00	0.60±0.62	1.00	0.00		1.00	0.431
	
P**(effect of time) pain	0.001									
	
P** (effect of group) pain	0.283									
P** (group and time interaction) pain	0.453					Observed power=0.274 Partial eta-squared (Effect size)=0.015				

* Mann-Whitney U test; ** Repeated measures analysis of variance

The trends of nausea and vomiting variations were not significant at the measurement times of 0 to 24 h in the acetaminophen (P=0.098) and gabapentin (P=0.675) groups. The two groups had no significant difference regarding nausea and vomiting at measurement intervals (P=0.897). According to the measurement times, no significant difference was observed in the levels of nausea and vomiting between the two groups. Furthermore, the trends of nausea and vomiting variations were not significant in both studied groups during the measurement intervals (P=0.582; Table .3). 

In the current study, the highest level of morphine administration was reported in the gabapentin group at the time of 0 after the operation (66.7% and 63.3%). In addition, the highest level of ondansetron administration was also reported in the 6^th^ hour following the surgery (13.3% and 10%).

**Table 3:** Postoperative nausea and vomiting in two studied groups

**Time interval following surgery**	**Gabapentin group**				**Mean±standard deviation**	**Median**	**P 25**	**P 75**	**P-value**
	**Mean±standard deviation**	**Median**	**P 25**	**P 75**					
Nausea and vomiting at 0 hour	0.43 ± 1.14	0.00	0.00	0.00	0.53 ±1.33	0.00	0.00	0.00	0.726
Nausea and vomiting at 2^nd ^hour	0.50±1.20	0.00	0.00	0.00	0.43±1.07	0.00	0.00	0.00	0.766
Nausea and vomiting at 4^th ^hour	0.60±1.54	0.00	0.00	0.00	0.83±1.58	1.00	0.00	0.00	0.282
Nausea and vomiting at 6^th ^hour	0.53±1.36	0.00	0.00	0.00	0.83±1.66	1.00	0.00	0.00	0.370
Nausea and vomiting at 12^th ^hour	0.70±1.53	0.00	0.00	0.00	0.40±0.86	0.00	0.00	0.00	0.826
Nausea and vomiting at 24^th ^hour	0.27±0.78	0.00	0.00	0.00	0.17±.38	0.00	0.00	0.00	0.954
P** (effect of time) nausea and vomiting	0.098				0.675				
P** (effect of group) nausea and vomiting	0.897								
P** (group and time interaction) nausea and vomiting	0.582				Partial eta-squared (Effect size)=0.012 Observed power=0.221				

* Mann-Whitney U test; ** Repeated measures analysis of variance

The general linear model was used for the comparative evaluation of the use of acetaminophen suppository and gabapentin suspension and levels of nausea and pain by controlling age, weight, duration of surgery, as well as the use of analgesic and antinausea drugs. There was no significant difference regarding the effects of the two drugs (i.e., acetaminophen suppository and gabapentin suspension) on the severity of pain with the control of confounding variables (i.e., age, weight, surgical duration, as well as the use of analgesic and antinausea drugs) (P=0.731). 

There was only a significant difference regarding the use of analgesics (P<0.001) and interaction between the treatment group receiving analgesic (P=0.026) and antinausea drugs (P=0.04). In other words, the effect of analgesic and antinausea drugs was different in both groups. The effects of studied drugs (i.e., acetaminophen and gabapentin) on nausea was not statistically significant with the control of confounding variables (P=0.115).Only the use of antinausea drugs (P<0.001), duration of surgery (P=0.005), and weight (P=0.05) had significant effects on nausea. The interaction of the type of drugs and use of analgesic (P=0.420) and antinausea drugs (P=0.113) was not significant on the level of nausea based on the generalized estimating equation. 

## Discussion

Pain and problematic swallowing following tonsillectomy are among the worst conditions for management in otolaryngology (10). Pain management in children is one of the most important healthcare challenges, and children, especially compared to adults, are more strongly affected by the adverse effects of postoperative pain (1) or complications of using analgesics (9). In the present study, the comparison of pain severity based on the VAS scores between the two groups during the studied times showed there was no statistically significant difference at any time. The pain severity in both groups was reported with a statistically significant decreasing trend; however, the trend of pain changes in two studied groups was not significant in the measurement times. In a study carried out by Amani and Abedinzadeh, they compared the effects of oral gabapentin with the IV injection of pethidine and local injection of bupivacaine. Based on the results, the gabapentin and bupivacaine groups were reported with the lowest and the highest pain scores, respectively. The pain score in the gabapentin group was significantly lower than that reported for the bupivacaine group, and there was no significant difference in the pain scores between the pethidine and bupivacaine groups (12). 

In another study conducted by Mogadam et al. (8), it was reported that the administration of oral gabapentin and diclofenac suppository before the operation could significantly reduce postoperative pain, compared to that in the control group. Similar to the results of the present study, this decrease was higher in the gabapentin group, but with no significant difference. The results of the aforementioned study showed that despite the effectiveness of both drugs in postoperative pain, the patients in the diclofenac group used analgesics for a longer period, indicating the long-term effect of gabapentin, compared to that of diclofenac. The two drugs also reduced the consumption of opioids without any clear adverse effects. However, in the current study, the comparison of both groups at the follow-up intervals demonstrated that the highest level of morphine administration was reported in the gabapentin group at 0 h after the surgery (66.7% and 63.3%), and a lower level of opioids was used in the acetaminophen group. 

In the present study, only the effects of the two drugs were compared 24 h after the surgery; nevertheless, in a study carried out by Mikkelsen et al. (13), the effects of gabapentin before and after the operation were compared with those reported for the placebo group. Although the severity of pain was similar in the two groups, in contrast to the findings of the current study, opioid use in the gabapentin group was significantly less than that in the placebo group. In the aforementioned study, the patients received a 1200, 1200, and 1800 mg doses of gabapentin before the surgery, during 24 h after the operation, and during 5 days after the surgery, respectively; however, in the present study, the children received only a 10 mg/kg dose of gabapentin syrup 2 h before anesthesia. 

In the above-mentioned study, the patients in both groups received a 50 mg dose of rofecoxib before and after the surgery (during 5 days of follow-up). This was an NSAID that can affect postoperative pain, and no difference between the two groups in terms of pain might be due to the use of this drug in two groups. Similarly, in a study performed by Mogadam et al. (8), the administration of diclofenac as an NSAID could lead to no difference in gabapentin and diclofenac groups regarding. In the aforementioned study, the cumulative pain score during swallowing 50 ml of water was observed in the group not receiving gabapentin; however, the tendency was not significant. 

In the above-mentioned study, in the gabapentin group, the required mean dose of meperidine was about 15 mg, and the onset of oral intake was approximately 2 h earlier. In the above-mentioned study, blinding to treatment allocation was not been reported which could lead to bias, and it is unclear whether patients complained about receiving postoperative meperidine spontaneously or due to pain. This is important because gabapentin might reduce the demand for analgesics by increasing its postoperative sedation effect, not due to its pain reduction effect. Given that the sedation was not evaluated in the above-mentioned study, its role was not clear in the patients’ demand for analgesics. 

In another study conducted by Jeon et al. (14), the patients received a preoperative dose of 600 mg gabapentin, and the use of opioids and pain severity was compared to those reported for the patients in the placebo group. The subjects with postoperative pain in two groups received diclofenac if needed. Based on the results of the aforementioned study, a significant decrease was observed in dynamic pain in the first 2 h after the operation; nevertheless, there was no difference between the two groups regarding the resting pain scores. They also showed a significant decrease in the use of opioids after surgery, compared to that reported for the placebo group. However, a meta-analysis carried out by Hwang et al. (10), focused on the analysis of studies comparing the gabapentinoids group (i.e., gabapentin and pregabalin) with the placebo or a pain control agent during a 24-hour period after the surgery. It was indicated that the pain scores reported by physicians during the first 8 h and need for the use of analgesics during the 24 h after the surgery significantly decreased in the patients receiving gabapentin, compared to those reported for the control group. The observed difference between the current study and other studies might be due to the comparison of gabapentin and acetaminophen, which could lead to no difference between the two groups in terms of their effects on pain reduction. 

It has been shown in various studies that the drugs, such as gabapentin and pregabalin, not only decrease the severity of acute pain and required doses of opioids but also can play a role in the prohibition of the chronic postoperative pain. In a systematic review carried out by Hwang et al., it was demonstrated that the use of gabapentin before surgery can reduce the administered dose of opioids and associated complications (10). 

Gabapentin might also play a role in the prevention and development of chronic postoperative pains and reduction of perioperative anxiety (15). It seems that gabapentin could be effective through interaction with the α2δ subunit of voltage-dependent neuronal channels, and its adverse effects, including sedation and dizziness, appear to be associated with the central nervous system. Since gabapentin mechanism of action does not include the coagulation system, it does not seem to have an effect on bleeding complications and might reduce the level of postoperative nausea and vomiting (PONV), probably due to its opioid inhibitory effect (9,16). It has been shown that gabapentin reduces other adverse effects associated with opioids, such as pruritus (16).

In the present study, the incidence of nausea and vomiting during postoperative periods (0 to 24 h) was reported with various fluctuations between the measurement times in both groups. The highest level of PONV was in the gabapentin and acetaminophen groups 12 and 4-6 h after the consumption, respectively. However, these differences were not significant in both groups at time intervals. Moreover, no significant difference was observed in the levels of nausea and vomiting between the two groups based on the time of measurement. The trend of changes in nausea and vomiting was not significant between the two study groups during the measurement times. 

In a study carried out by Mogadam et al. (8) on the adverse effects, such as nausea, dizziness, and headache, no significant difference was reported between gabapentin, diclofenac, and placebo groups. In a meta-analysis performed by Hwang et al. (10), the incidence of nausea and vomiting during postoperative periods (0 to 24h) was significantly lower in the gabapentinoids group than that reported for the control group. Moreover, in the aforementioned study, the levels of sedation during these measurement periods were not significantly higher in the gabapentinoids group than those reported for the control group. According to the results of the aforementioned study, gabapentinoids do not increase the adverse effects after the tonsillectomy (9). 

The results of a study conducted by Mikkelsen et al. also showed that gabapentin reduced the need for opioids in the first 24 h after tonsillectomy; however, it induced more dizziness (3 times), vomiting (5 times), and walking disorders (4 times), compared to those reported for the placebo group on 0 to 5 days after the surgery. Nonetheless, the differences in this regard were statistically significant (13). These adverse effects might be due to receiving a preoperative 1200 mg dose of gabapentin and daily 1800 mg dose for 5 days after the surgery. 

In a study conducted by Mikkelsen et al., the benefits of decreasing opioid use through gabapentin might be affected by adverse effects. In a study carried out by Jeon et al. (14), no complications (e.g., nausea, vomiting, sedation, or dizziness) were reported in the gabapentin group. In the current study, the highest level of ondansetron administration was observed in the gabapentin group 6 h following the surgery (13.3% and 10%). In addition, the gabapentin group had a lower level of PONV than that reported for the control group. The level of decreasing PONV was higher in the gabapentin group, but with no significant difference.

In the present study, although the differences in the weight and age of the children were statistically significant between the two groups and the subjects in the gabapentin group were approximately 3 kg heavier and 2 years older than those in the acetaminophen group, these differences did not affect the result of the study. The reason is that the effects of acetaminophen suppository and gabapentin suspension on the pain severity with the confounding variables of age, body weight, duration of surgery, use of analgesics and anti-emetic drugs were not significant. Furthermore, the effects of two drugs on nausea were not statistically significant with controlling the studied confounding variables. 

In a study carried out by Mogadam et al., there was no statistically significant difference between the age, gender, and duration of surgery in the patients in three gabapentin, diclofenac, and placebo groups (8). In another study performed by Mikkelsen et al. (13), no significant difference was observed in the weight, age, mean dose of sufentanil or alfentanil, and duration of surgery between the two groups. 

Acetaminophen is the most common analgesic used for children. It is an effective analgesic and antipyretic with similar power to aspirin for the treatment of mild to moderate pains. In contrast to aspirin, acetaminophen does not inhibit the function of platelets leading to less intestinal disorder and no Reye's syndrome (17). A study carried out by Haddadi et al. compared the effects of IV acetaminophen and acetaminophen suppository on the reduction of postoperative pain in 96 children within the age range of 4-10 years after adenotonsillectomy. According to the results, it was reported that the level of pain severity was significantly lower in the group receiving acetaminophen suppository after the surgery. In the group receiving IV acetaminophen, only 10.4% of the patients were reported with no pain; however, in the group receiving rectal acetaminophen, this rate reached 43.8%, and the demand for analgesics in the rectal acetaminophen group was significantly lower than the IV group (18). 

Although the results of the present study showed that there was no significant difference between the acetaminophen suppository at the dose of 40 mg/kg and gabapentin syrup at the dose of 10 mg/kg in terms of pain relief and antinausea and vomiting effects, the use of NSAIDs increases the risk of postoperative bleeding. In addition, the administration of opioids increases the risks of PONV and respiratory depression. Acetaminophen alone provides a poorer pain decreasing effect than opioids, and patients require more rescue analgesia (8). The results of some studies have shown that codeine/acetaminophen is not superior to acetaminophen alone regarding pain control (3). In most clinical trials conducted on the effectiveness of gabapentinoids (i.e., gabapentin and pregabalin) in postoperative pain relief, it was reported that the pain score and need for analgesics during 24 h after surgery significantly reduced in the patients receiving gabapentin, compared to those reported for the control group. Furthermore, it was indicated that gabapentin can cause this pain without adverse effects and with a reduced need for opioids (10). 

Based on the literature, there has been no study carried out on the respiratory depression caused by gabapentin (16). The difference between the present study and other investigated studies was the use of low-dose gabapentin in the current study and higher dose of this drug in other studies (8,13-16,19). The current study examined posttonsillectomy pain, nausea, and vomiting with the prescription of low-dose gabapentin in addition to decreasing the side effects and sedation resulting from gabapentin. One of the limitations of this study was obtaining a sufficient sample size due to the age range.

## Conclusion

The obtained results of this study showed that the preoperative administration of low-dose gabapentin can reduce the severity of pain in the patients undergoing adenotonsillectomy without any adverse effects. The effects of acetaminophen suppository and gabapentin suspension on pain severity and nausea with the control of the confounding variables of age, weight, duration of surgery, and use of analgesic and antinausea drugs were not significant. 

However, due to the adequate evaluation of the effectiveness of the gabapentinoids and given the high heterogeneity in some parameters, it is recommended to perform further clinical trials with a robust research design. The reason is that gabapentin might reduce the demand for analgesics by increasing the effect of sedation after surgery, not due to the effect of pain reduction, and the sedation should be evaluated in future studies. As a result, the role of gabapentin in the requests for analgesics should be clarified in order to confirm the results of the present study. It is also suggested to investigate the severity of postoperative pain at the time of swallowing or resting during the first 24 h after surgery using both approaches. It is also recommended to carry out further studies with larger sample sizes, determination dynamic or resting pain, and response to treatment.
